# Optimising co-design with ethnic minority consumers

**DOI:** 10.1186/s12939-021-01579-z

**Published:** 2021-11-04

**Authors:** Ashfaq Chauhan, Jessica Leefe, Éidín Ní Shé, Reema Harrison

**Affiliations:** 1 Centre for Health Systems and Safety Research, Australian Institute of Health Innovation (AIHI), Macquarie University, North Ryde, NSW 2109 Australia; 2 System Transformation Evaluation and Patient Experience, Agency for Clinical Innovation (ACI), NSW Health, St Leonards, NSW 2065 Australia; 3 School of Population Health, University of New South Wales (UNSW), Sydney, NSW 2052 Australia

**Keywords:** Ethnic minorities, Co-design, Seldom heard, Equity, Consumer co-leadership

## Abstract

Co-design as a participatory method aims to improve health service design and implementation. It is being used more frequently by researchers and practitioners in various health and social care settings. Co-design has the potential for achieving positive outcomes for the end users involved in the process; however, involvement of diverse ethnic minority population in the process remains limited. While the need to engage with diverse voices is identified, there is less information available on how to achieve meaningful engagement with these groups. Ethnic minorities are super-diverse population and the diversity between and within these groups need consideration for optimising their participation in co-design. Based on our experience of working with diverse ethnic minority groups towards the co-design of consumer engagement strategies to improve patient safety in cancer services as part of the two nationally-funded research projects in Australia, we outline reflections and practical techniques to optimise co-design with people from diverse ethnic backgrounds. We identify three key aspects of the co-design process pertinent to the involvement of this population; 1) starting at the pre-commencement stage to ensure diverse, seldom heard consumers are invited to and included in co-design work, 2) considering logistics and adequate resources to provide appropriate support to address needs before, during and beyond the co-design process, and 3) supporting and enabling a diversity of contributions via the co-design process.

## Main text

### Co-design in healthcare research and practice

While not a new philosophy, the use of co-design as a participatory method to help improve health services design and implementation has piqued interest of policy makers, health service providers, government health agencies along with health service researchers in recent years in the context of promoting person-centric care [1]. Co-design and associated methodologies such as co-production, co-research and co-creation, have been used by researchers and practitioners in various health settings, although applications of the process of co-design vary [2–4]. Fundamental to co-design, is the bringing together of consumers and health service and system stakeholders to design or adapt the design of, processes and programs based on the lived experiences of end users (most commonly health consumers and staff) [5].

Although co-design has potential for achieving positive outcomes for the included end users [6], those excluded from co-design processes may not reap such benefits [7]. Often, super-users (users who frequently contribute to research projects) are invited to participate in co-design projects due to convenience, their understanding of the processes and role, their ability to articulate, and their ability to contribute to the project [8, 9]. This group of super-users may not represent the whole of the population that is accessing the health service [10]. Seldom heard groups are described as groups who may experience barries in participation in healthcare decision-making or accessing healthcare services [11]. Seldom heard groups can include people from ethnic minorities, refugees and asylum seekers, older people who are frail, people with intellectual disability, people with mental health issues, rural and regional communities, and gay, lesbian, bisexual, transgender and intersex people [9]. Existing organisational structures and practices for involvement may not provide opportunity for equal involvement with seldom heard groups as the current processes and structure may not be conducive to their needs for equal participation [11, 12]. An inability to consider the consequences arising from the lack or diverse representation in co-design may risk exacerbating existing biases experienced by seldom heard groups and or limit the uptake of co-designed change [13, 14].

### Diversity in co-design

The need for diverse representation within co-design, and to consider the processes, methods and tools that encourage and amplify diverse voices is therefore recognised [7, 14, 15]. Involving diverse participants in healthcare co-design can bring novel solutions to the issue at hand by encapsulating a broader variety of experiences and knowledge [16]. It can also work to ensure that changes resulting from co-design do not heighten inequities in care. Promoting the participation of seldom heard groups in co-design can however be challenging [17, 18]. Principles of co-design are focused on equal power distribution, building capacity and capability, building relationships, and promoting active participation [4, 5, 14]. Values-based approaches to co-design identify respect, openness, reciprocity, and flexibility as critical from the pre-commencement stage of co-design to promote equal participation [19].

Resources and research principles have been developed to address inequities in co-design participation for key populations such as young people, those with disability and in the context of mental health. For example, frameworks and toolkits that enhances involvement and experiences of the disadvantaged and vulnerable population in co-design process have been proposed [9, 15, 20–23]. In their study using a modified case study approach to examine challenges against theoretical propositions, Mulvale et al., promotes enacting a set of principles rather than following a set of predefined steps when co-designing with populations such as adults and youths in mental health contexts, indigenous populations, and survivors of domestic violence [21]. A six-step framework proposed by Dietrich et al., based on their insights generated from six co-design groups with adolescents, comprised of resourcing, planning, recruiting, sensitising, facilitation and evaluation as six steps for enhancing involvement of adolescents in co-design process [22]. Despite recognition of the need to ensure ethnic diversity involvement within engagement practices more generally in healthcare, supporting by a recent document analysis of engagement frameworks in Australia, the mechanisms of how to achieve strong engagement in healthcare generally and resources to support the operationalisation of inclusive co-design with ethnic minority populations specifically remain limited [12].

### Amplifying contributions from ethnically diverse consumers

People from ethnic minority backgrounds are often recognised as being exposed to health inequalities due to minority status, health inequities and cultural and/or language barriers [24]. Ethnic minorities are a super-diverse group [25]: with differing levels of educational background, professions, reasons for migration, levels of language proficiency, migration experiences and cultural and/or religious and spiritual practices. These factors independently and collectively influence their contribution and support requirements to participate in co-design [26]. Yet inter- and intra-group diversity is often overlooked and critical to consider for ensuring diverse perspectives are introduced and supported in co-design [24].

While the INVOLVE (lead for enhancing public involvement in health and care research across National institute of Health Research) report provide an overview of key principles and guidelines for an optimal co-design [27], guidance on practical adoption of the principles of co-design with ethnic minority consumers is lacking. Based on our experience of working with diverse ethnic minority groups (people that have specific cultural or linguistic affiliation by virtue of their place of birth, religion, preferred language, or language spoken at home) in Australia to co-design patient engagement strategies for patient safety in cancer services as part of the two nationally-funded research projects, we outline practical techniques to optimise co-design with this super-diverse group. These practical techniques and approaches are informed by a two-year research process and the contributions of the CanEngage project team, consumer advisory group, and the project steering group.

### Practical techniques and approaches

**Fig. 1:**
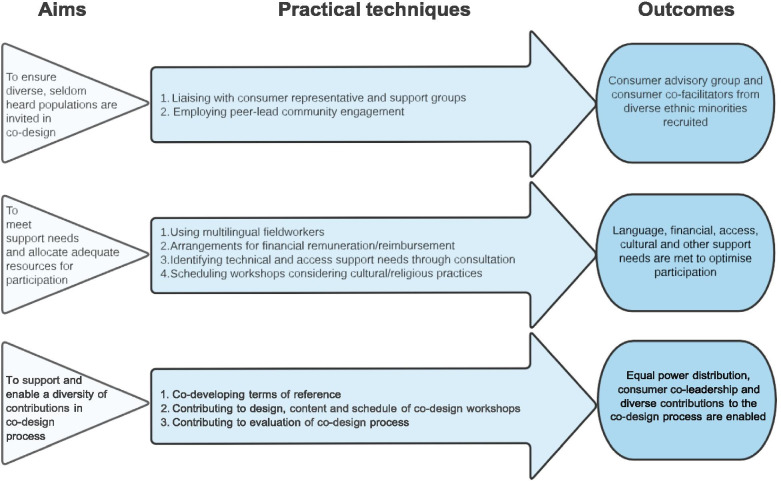
Practical techniques and intended outcomes in optimising co-design with ethnic minority consumers

We reflect upon three key areas in planning and conducting co-design with ethnic minority consumers to optimise the process and its potential to lead to the desired outcomes: firstly ensuring diverse, seldom heard consumers are invited to and included in co-design work, secondly providing appropriate supports to address needs before, during and beyond the co-design process, and finally supporting and enabling diverse contributions as part of the co-design process (Fig. 1).

1. *Start at the pre-commencement stage to ensure diverse, seldom heard consumers are invited to and included in co-design work:*

Engaging with relevant community groups and members is an important step preceding co-design for meaningful engagement with the community to determine community needs and priorities [9]. At the outset of our projects, our team sought to identify individual community members to participate in the co-design projects and to bring together a diverse consumer advisory committee to guide the project through its lifecycle. In seeking individual community members for the co-design, we identified a wide variety of community groups and networks across the state of New South Wales and also nationally in Australia. We spoke with consumers from a wide variety of ethnic and linguistic groups, in addition to cancer services and support organisations in many locations to establish the groups and networks available. One of the critical opportunities created by this approach to community engagement was to open up opportunities to know about and become involved in co-design from its inception for consumers beyond those who are closely engaged with the health system and healthcare research. Peer-led community groups were particularly important in this regard to reach those for whom English was not their first language or who were not closely connected to the health system through their networks [9, 28]. Identifying consumers through peer led community groups is described as an approach that promotes trust within research processes [28–30]. Working through peer-led community groups meant that prospective members could work within existing relationships of trust and rapport to approach potential co-design members appropriately, for them to ask questions about the project, identify their specific needs and discuss concerns. We spoke with cancer-specific community groups that provide support to ethnic minority consumers to identify consumers to invite to join the consumer advisory committee, resulting in five members from a diverse range of backgrounds and with lived experience of cancer. The role of the consumer advisory committee was to inform, guide and influence every aspect of the projects and help us to navigate the approach to consumer engagement throughout. The advisory committee members were embedded in wider community networks and this provided a highly -valued resource to support the team to identify and recruit consumer co-facilitators from diverse ethnic backgrounds to co-lead the co-design process.

2) *Logistics and adequate resources to provide appropriate support to address needs before, during and beyond the co-design process:*

Before commencing co-design, it is essential to identify what supports participants may require and how these can be ensured before, during and beyond co-design process. Ethnic minority consumers have a diversity of needs, some of which may reflect those of mainstream health service users. In our projects, initial consultation with our diverse consumer advisory group of end users with lived experiences identified a range of potential needs for service users from diverse ethnic minorities that we should consider ahead of embarking on the program of work. These needs included providing language support, financial support, access support (technical and other), additional time to process and respond to tasks during the co-design process and the need to consider the religious and cultural calendars and traditions of those involved when planning meetings. Multilingual fieldworkers were employed to support some of these requirements to navigate such needs along with addressing the language barriers [26]. For example, in planning the timings of the co-design workshops, we reviewed and considered the cultural or religious practices observed by the participants to ensure that the date, time and session configuration did not prevent them from participation. We have recruited and trained multilingual fieldworkers in previous research and found this method to be effective in increasing the quality of engagement within group processes for a range of ethnic minority groups [26]. We addressed the potential for language support beyond translation or the use of multilingual facilitators by using plain language for conversation, removing acronyms and being aware of the significance of specific terms. The ethnic diversity within our research team and supported by the diverse consumer co-facilitators further enabled us to reflect upon nuanced cultural differences that exist between diverse ethnic groups and attempt to plan our activities accordingly.

Financial remuneration and reimbursement are identified as an important step to support participation of consumers in research; this is also relevant to health service improvement activities [21]. Yet financial remuneration can also pose challenges for consumers and preferences vary as to the mode of renumeration; ensuring a suitable mode of renumeration is therefore critical [31]. In developing the Terms of Reference for our co-design groups, we asked participants to indicate individually and confidentially as to whether they would like to be reimbursed for costs incurred and remunerated for their time (gift vouchers, charity donation etc.) We then made the arrangements as requested for by each consumer co-facilitator and co-design member. Developing the Terms of Reference in collaboration with the participants (consumers and service providers) and consumer co-facilitators prior to the co-design workshops, and maintaining this as a dynamic document, allowed us to agree the duration and location/mode for the workshops (hybrid, online or face to face), the online platforms to be used when hybrid or online workshops were being conducted, and to agree when materials (translated or otherwise as required) would be received (generally at least 1 week prior to the workshops). For face-to-face workshops, arrangements will be made such that the workshops are located in a neutral space (outside of the hospital setting) and the appearances and dress codes kept neutral (avoid wearing hospital uniforms or suits) to promote equal power distributions between the participants.

3) *Support and enable a diversity of contributions* via *the co-design process:*

Equal power distribution is a key principle for co-design. Failure to address power imbalance between service providers, researchers and service users may lead to loss in trust in the service and the co-production approaches resulting in more harm than good [21, 32]. We sought to address this issue in part by recruiting and training consumers as co-facilitators. Drawing upon our deep-seated links with the community, we advertised for, recruited and train nine consumer co-facilitators and multilingual fieldworkers to support the co-design process. We developed role descriptions for the consumer co-facilitators and multilingual fieldworkers with payment made for their time as per the guidance provided by Health Consumers NSW [33]. Opportunities to contribute to the project were made clear with participation sought in identifying the safety issues for focus, co-leading the workshops, planning the workshops, assisting with CALD consumer engagement in workshops, and liaising with participants in between workshops to collect their feedback. Consumer co- facilitators took a leadership role, making decisions on how the sessions will be run, moderated and their content [12]. The scope of this role was collaboratively refined in developing the Terms of Reference. Including a consumer co-lead provided a mechanism to reflect on and address implicit biases in the co-design process. Consumer co-facilitators as co-lead also promoted consumer leadership whereby they are recognised as decision-makers within the process to advance consumer participation within the co-design process [10]. Consumer participants and facilitators as the majority members within the workshops also elevated their opportunity to contribute comfortably. The need to provide an opportunity for co-design members to provide feedback to the process in between the workshops due to the time needed to consider and process information also emerged. A process was therefore established whereby the consumer co-facilitator would liaise with the consumer co-design members individually, via phone/online applications/online medium as preferred by each participant, in between workshops to collect their feedback on the items discussed. Consumer co-facilitators and multilingual fieldworkers have been invited to contribute to the process and outcome (summative and formative) evaluation of the co-design process and advised of opportunities to become involved in development of manuscripts for publication, and co-presenting research outputs with the research team as optional activities.

The success of the approaches outlined are contingent upon health system environment, culture and organisational commitment that prioritises and sufficiently resources high-quality co-design [7]. Commitment from senior leadership and management is required along with making high-quality co-design a strategic priority, involving decision-makers in co-design process, and allocating funds at the outset [7, 34].

### Conclusion

Improving equity in healthcare delivery and outcomes for people from ethnic minority backgrounds is promoted through inclusive approaches to health services research and improvement. Ensuring adequate supports and group processes in co-design ensures that diverse voices are included and heard within the process and are able to influence the outcomes to address issues of importance to this population. Consideration of individual and group needs is critical towards this. Developing strong connections with ethnic minority communities, partnering with and training consumer co-facilitators to co-lead the workshops, addressing socio-cultural needs of the participants and collaboratively developing Terms of Reference are some approaches that we have found to be valuable to support involvement of ethnic minority groups by addressing issues of power imbalance, to enhance individual’s experiences of participation and their ability to influence the process and outcomes for better care.

## Data Availability

Data sharing not applicable – no new data generated.
